# Knowledge, Attitudes and Behaviors regarding Tick-Borne Encephalitis Vaccination and Prevention of Tick-Borne Diseases among Primary Care Physicians in Bavaria and Baden-Wuerttemberg, Germany, May–September 2022

**DOI:** 10.3390/microorganisms11040961

**Published:** 2023-04-07

**Authors:** Liza Coyer, Aylin Sogan-Ekinci, Benedikt Greutélaers, Julia Kuhn, Franziska S. Saller, Jana Hailer, Stefanie Böhm, Rainer Brosch, Christiane Wagner-Wiening, Merle M. Böhmer

**Affiliations:** 1 Infectious Disease Epidemiology and Surveillance Unit, Bavarian Health and Food Safety Authority (LGL), 80636 Munich, Germany; benedikt.greutelaers@lgl.bayern.de (B.G.); franziska.saller@lgl.bayern.de (F.S.S.); stefanie.boehm@lgl.bayern.de (S.B.); 2 ECDC Fellowship Programme, Field Epidemiology Path (EPIET), European Centre for Disease Prevention and Control (ECDC), 16 973 Solna, Sweden; 3 Department of Health Protection, Infection Control and Epidemiology, Baden-Wuerttemberg Federal State Health Office (LGA), Ministry of Social Affairs, Health and Integration, 70191 Stuttgart, Germany; aylin.sogan-ekinci@sm.bwl.de (A.S.-E.); julia.kuhn@sm.bwl.de (J.K.); rainer.brosch@sm.bwl.de (R.B.); christiane.wagner-wiening@sm.bwl.de (C.W.-W.); 4 District Health Office Reutlingen, 72764 Reutlingen, Germany; jana_hailer@kreis-reutlingen.de; 5 Faculty of Medicine, Ludwig-Maximilians-University of Munich, 80336 Munich, Germany; 6 Institute of Social Medicine and Health Systems Research, Otto-von-Guericke-University, 39120 Magdeburg, Germany

**Keywords:** tick-borne diseases, tick-borne encephalitis, TBE, Lyme borreliosis, tularemia, prevention, vaccination, education, physicians

## Abstract

In 2020, a record number of tick-borne encephalitis (TBE) cases was reported in major endemic areas in Germany, i.e., the southern federal states of Baden-Wuerttemberg and Bavaria. Most cases were unvaccinated. Other tick-borne diseases (TBDs), including Lyme borreliosis and tularemia, are rising, too. Thus, strategies are needed to increase TBE vaccination uptake in risk areas and promote education on TBD prevention. Primary care physicians are key providers of both vaccinations and TBD education. The TBD-Prevention (TBD-Prev) study aimed to investigate the knowledge, attitudes and behaviors of primary care physicians in Baden-Wuerttemberg and Bavaria with regard to TBE vaccination and prevention of TBDs and to derive strategies for increasing vaccination rates and improving knowledge about TBE and other TBDs in the population and among primary care physicians. We invited all primary care physicians (N = 14,046) in both states to participate by mail. Using standardized, self-administered questionnaires, available both on paper and online, we asked physicians anonymously about their knowledge, attitudes and behaviors with respect to TBE vaccination and TBD prevention and their need for further information/educational materials. A total of 2321 physicians participated between May and September 2022 (response rate 17%), of whom 1222 (53%) worked in Baden-Wuerttemberg and 1067 (46%) in Bavaria. Among the participating physicians, 56% were male, 71% were >50 years and 51% worked in an individual practice. Furthermore, 91% were aware of the German national vaccination guidelines, and 98% perceived their knowledge of the risks and benefits of vaccination as adequate. A total of 97% offer TBE vaccinations, 67% provide vaccination counselling during initial consultations with new patients and 64% actively remind patients about due vaccinations. In addition, 24% expressed a need for further information materials, mainly traditional, analogue media such as flyers (82%) and posters (50%), and named timeliness, quality assurance, easy comprehensibility and independence from the pharmaceutical industry as the most important characteristics of such materials. Almost all participating physicians reported offering TBE vaccinations and feeling well-informed about TBE vaccination and TBDs. However, active offering of vaccinations and education could be further improved, and additional, low-threshold information materials are needed. Based on these results, we will develop and provide various materials on TBE vaccination and TBDs, in particular flyers and posters, for use by physicians during consultations.

## 1. Introduction

Tick-borne diseases (TBDs) are a significant public health concern, with several species of ticks capable of transmitting a variety of pathogens to humans. The most common TBD in Germany is Lyme borreliosis. Lyme borreliosis is a prevalent disease in the northern hemisphere caused by the spirochetes of the *Borrelia burgdorfi* sensu lato complex through the bites of infected *Ixodes* spp. ticks [1,2]. In the German federal state of Bavaria, 35,458 cases were reported between 2013 and 2020 [3]. In the absence of a mandatory notification, case numbers of Lyme borreliosis in the federal state of Baden-Wuerttemberg are unknown. Approximately 90% of Lyme borreliosis cases develop a characteristic skin rash (erythema migrans) within days to weeks after exposure [4]. Some cases simultaneously develop flu-like symptoms. Lyme borreliosis is treatable with antibiotics; however, in the absence of treatment, severe manifestations such as neuroborreliosis or Lyme arthritis can occur.

Tick-borne encephalitis (TBE) is the second most common TBD in Germany and is a viral infectious disease of the central nervous system caused by the TBE virus (TBEV) [5,6,7]. TBEV is endemic in Central, Eastern and Northern Europe, Russia and parts of China and Japan [5]. The most common TBEV subtype circulating in Europe is the European subtype (TBEV-Eu), which is transmitted by the hard-bodied tick species *Ixodes ricinus* (castor bean tick). Approximately one-third of TBEV infections are symptomatic [6]. The course of the disease can be biphasic. Non-specific symptoms including fever and malaise occur first, possibly followed by several neurological manifestations, including uncomplicated meningitis, meningoencephalitis and myelitis [8,9]. Approximately 10–20% of individuals infected with the European TBEV subtype (TBEV-Eu) develop second-phase symptoms [6,9,10]. While the overall mortality rate lies between 0 and 1.4%, around 25–50% of TBEV-Eu cases develop long-term effects [6]. There is no effective treatment for TBE, but anti-inflammatory medications are available for symptom relief. Between 2001 and 2022, 8192 acute TBE cases were reported in Germany [11]. Almost 90% of cases occurred in Bavaria and Baden-Wuerttemberg. TBE is notifiable in Germany since 2001 [12]. The TBE case definition thereby comprises both disease courses of a TBE infection with only unspecific, influenza-like symptoms, as well as neurological manifestations (meningitis, encephalitis, myelitis). As of March 2023, 94 of 96 administrative districts of Bavaria and 43 of 44 districts of Baden-Wuerttemberg are declared as TBE risk areas by the Robert Koch Institute (national public health institute in Germany) [13]. The annual number of reported TBE cases in Germany has increased over time from 255 cases in 2001 to a record of 717 cases in 2020, with particularly high case numbers since 2017 [11]. More than 90% of cases occur annually between May and October, although cases are reported earlier each year, presumably because periods of tick activity tend to start earlier in the season and last longer due to climate change [14,15].

Furthermore, tularemia—caused by the bacterium *Francisella tularensis*—is an emerging disease, and increasing case numbers have been reported in Germany in recent years; the overall number of tularemia cases (tick-borne and other) reported in Bavaria and Baden-Wuerttemberg increased from 3 in 2001 to 64 in 2021 [16]. There is no vaccine available against tularemia to date, but antibiotics can relieve symptoms. Infected persons often develop influenza-like symptoms, followed by more specific symptoms which can be categorized into seven different clinical syndromes, depending on the site of infection. In the early stage, tularemia mainly presents with non-specific, influenza-like symptoms. In a progressed stage of disease, the most common form is the ulceroglandular/glandular form, which is caused by contact with contaminated animal material or water, via skin lesions or mucous membrane or via stings and bites of infected arthropods. The oculoglandular form is caused by touching the eye after contact with contaminated material or an infected animal. The oropharyngeal form is caused by the oral intake of contaminated food or water. Finally, pulmonal/respiratory forms are caused by the inhalation of contaminated dust or aerosols [17].

Prevention of tick bites is the most important strategy to prevent TBDs. Prevention measures include wearing closed-toe shoes, long sleeves, long trousers with trousers tucked into socks and bright clothes; using repellents; staying on paths; avoiding long grass and bushes; not touching wild animals; and checking for ticks after being outside (and removing ticks as soon as possible). In addition, safe and highly effective vaccines against TBE are available [18,19,20]. The German Standing Committee on Vaccination (STIKO) recommends TBE vaccination for all persons in Germany potentially exposed to ticks in TBE risk areas [21]. Health insurance covers costs for such recommended vaccinations. Despite this, vaccination uptake is very low, even in risk areas (Bavaria: 22%; Baden-Wuerttemberg: 18%) [13]. Moreover, TBE vaccination uptake has been decreasing over time [22]. It is thus important to develop strategies to improve vaccination rates in order to prevent incident TBE cases. The key targets of such strategies are primary care physicians, who are the main providers of vaccinations in Germany. Studies consistently show that physician recommendations positively influence clients’ decisions to receive vaccination [23,24,25]. Primary care physicians do not only have the necessary expertise, but are also trusted, as doctor–client contacts have often been built up over years. Moreover, they are first-line care providers for most patients and are well-positioned to monitor vaccination status, provide vaccination counselling, send reminders of due vaccinations and provide vaccinations when needed. Primary care physicians are also key providers of general advice on the prevention of TBDs.

Increased uptake of TBD prevention strategies, including TBE vaccination, in Bavaria and Baden-Wuerttemberg could prevent a large proportion of TBE cases and other TBDs. However, there is insufficient information from Bavaria and Baden-Wuerttemberg on the knowledge, attitudes and practices of primary care physicians in private practices with regard to TBE; other TBDs and measures for their prevention, including TBE vaccination; and the possible need for further or different information materials. As such, we implemented a survey among all primary care physicians in private practices in Bavaria and Baden-Wuerttemberg to derive strategies for increasing vaccination rates and improving knowledge about TBE and other TBDs in the population and among physicians.

## 2. Materials and Methods

### 2.1. Study Design and Population

From 24 May to 30 September 2022, we implemented a cross-sectional survey in the context of the TBD-Prevention (TBD-Prev) study among all primary care physicians working in private practices (which includes general medicine, internal medicine without further specialisation and pediatrics) in the German federal states of Bavaria and Baden-Wuerttemberg. Of the 14,046 invited physicians, 2333 responded.

### 2.2. Procedures

We sent the survey invitations by mail, seeing as a complete list of all practice addresses, but not email addresses, is available via State Medical Associations in Germany. Participants could self-complete the survey anonymously on paper (free-of-charge return was provided) or online through the platform Lamapoll [26]. We promoted the survey through regional medical journals (e.g., *Bayerisches Ärzteblatt*), professional societies, state medical associations and Local Public Health Authorities. The ethics committee of the Bavarian and Baden-Württemberg Medical Associations considered the study exempt from institutional review board approval. The Data Protection Officers of the Bavarian Health and Food Safety Authority and the Baden-Wuerttemberg Federal State Health Office additionally examined and approved the study.

### 2.3. Questionnaire Content

We asked physicians about their knowledge, attitudes and behaviors with respect to TBE vaccination and TBD prevention, and their need for further information and educational materials. The original questionnaire was in German. Both the German and translated English version can be found in Supplementary Materials S1 and S2. Specifically, we asked the following information:

- Demographic characteristics (i.e., gender, age, TBE and influenza vaccination status);
- General practice data (i.e., type of practice, license plate area code of practice, medical specialty). We used the license plate area code to determine the work state and classify urban–rural level according to the nomenclature of territorial units for statistics (NUTS) 2021 level 3 [27];
- Awareness of TBE guidelines and experience with TBE and other TBDs (i.e., awareness of STIKO recommendations, STIKO vaccination app and TBE risk area status, experience with treating TBDs, self-perceived adequacy of knowledge about risks and benefits of TBE vaccination);
- General vaccination and TBE vaccination practices (i.e., provision of vaccination to clients, method of conducting vaccination consultations, checking client’s vaccination status, reminding clients of due vaccinations);
- Reasons for not providing vaccinations (i.e., logistical difficulties, high effort for clarification, patient non-compliance, concerns about TBE vaccine safety, low perceived health risk of TBE, doubts about TBE vaccine effectiveness);
- Self-perception of adequate knowledge about risk and benefits of TBE vaccination;
- Specific knowledge needs and need for information materials (i.e., posters, information flyers, stickers, display stands, video clips, slide sets, infographs, others) on TBE vaccination, TBDs in general and tick prevention;
- Relative importance of different characteristics of information materials (i.e., independence of pharmaceutical companies, quality assurance, free-of-charge, up-to-date, easy to understand, specific to target group, accessibility, availability in different languages, others).

### 2.4. Statistical Analysis

We conducted a descriptive analysis. We described categorical data using frequencies, overall and by state (Baden-Wuerttemberg and Bavaria). We used R version 4.0.2 (Vienna, Austria) for data cleaning, analysis and visualization. Supplementary Material S3 lists the R packages that were used.

## 3. Results

### 3.1. Study Population

We received 1524 postal and 809 online responses (responses 2333/14,046; 17%), but 12 questionnaires contained only missing data and were removed from the analysis. Of the remaining 2321 responses, 1222 (53%) were from primary care physicians who worked in Baden-Wuerttemberg (response 1222/6750; 18%) and 1067 (46%) were from physicians who worked in Bavaria (response 1067/7296; 15%). For 1% (32/2321) of participating physicians, work state could not be determined. Table 1 reports the demographic characteristics and general practice data of the 2321 participating physicians. Briefly, 56% were male, 71% were older than 50 years, 51% worked in an individual practice, 66% indicated general medicine as their medical specialty and 81% indicated offering advice on travel medicine. The vast majority of physicians were vaccinated against influenza (85%) and TBE (84%). There were no major differences between states, though locations of practices were more rural in Bavaria.

### 3.2. Awareness of TBE Guidelines and Experience with Tick-Borne Diseases including TBE

Of 2321 participating physicians, 91% were aware of the German national vaccination guidelines, but fewer (34%) were aware of the corresponding vaccination app (Table 2). The vast majority was aware of TBE risk areas in Germany (90%) and that their practice was located in a risk area (99%). Almost all physicians had experience with treating Lyme borreliosis (96%), but fewer with TBE (48%), tularemia (2.4%) or other TBDs (8.6%). Furthermore, 98% perceived their knowledge of the risks and benefits of vaccination (in general) as adequate. There were no major differences in the awareness of and experience with TBDs based on state and urban–rural level, though experience with TBDs was slightly higher in more rural areas (as presented in Supplementary Material S4). Supplementary Material S5 reports the information needs among physicians who did not indicate having adequate knowledge. Education on vaccinations was the most indicated need, followed by training on vaccine-preventable diseases.

### 3.3. Vaccination Practices

Almost all (97%) physicians indicated providing vaccinations in general, and TBE vaccinations (97%) specifically (Table 3). Supplementary Material S6 presents the reasons for not offering TBE vaccinations. The most common method of offered vaccination consultations were in the context of preventive medical check-ups (89%) and at the active request of patients (80%). Moreover, 67% of physicians provided vaccination consultations during initial consultations with new patients, and 64% actively approached patients about due vaccinations. Most physicians (89%) regularly checked the vaccination status of patients and reminded them of due vaccinations (84%), including for TBE (79%), although fewer used a reminder system (28%). The most common method of reminding patients of due vaccinations was via cards/sheets listing their next vaccination (69%). There were no major differences between states.

### 3.4. Information Material Needs

**Figure 1:**
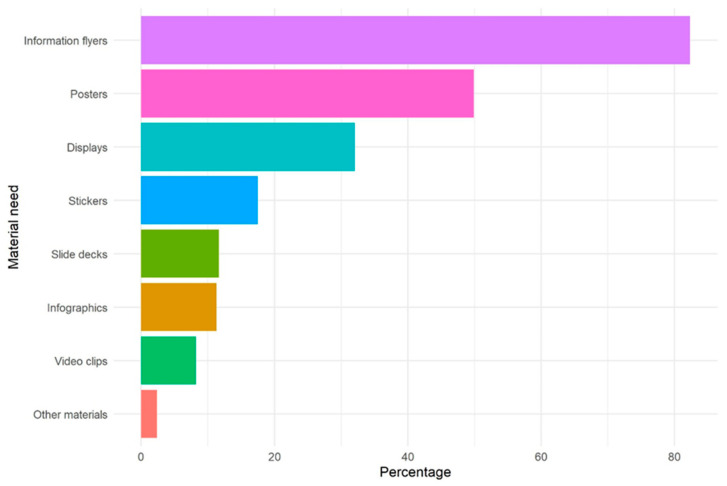
Type of information materials and educational media on TBE vaccination and tick-borne diseases needed, as indicated by primary care physicians with a need (N = 549), TBD-Prev survey, Germany, 24 May–30 September 2022. There were in total 558 physicians who indicated a need for information materials and educational media, but 9 did not complete the question about which kind of materials.

**Figure 2:**
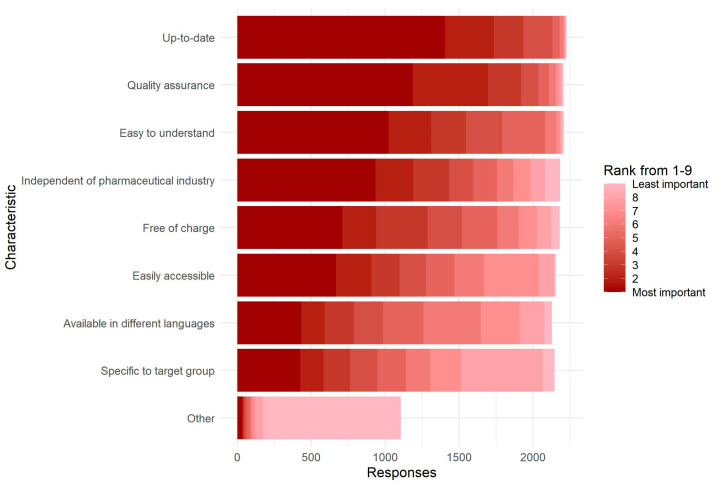
Characteristics of information materials about TBE vaccination and tick-borne diseases that are most important to primary care physicians (N = 2242), TBD-Prev survey, Germany, 24 May–30 September 2022. Physicians were asked to rank characteristics from 1 (most important) to 9 (least important). Of 2321 participating physicians, 79 did not answer this question.

A total of 71% of physicians indicated already using information materials on TBE vaccination and TBDs, mostly posters (76%) and flyers (76%), while 24% expressed a (further) need for information materials and educational media, mainly for traditional media such as flyers (82%) and posters (50%) (Figure 1). Physicians ranked timeliness, quality assurance, easy comprehensibility and independence from the pharmaceutical industry as the most important characteristics of such materials, without much difference between physicians with and without a further need for these materials (Figure 2). There was no major difference in the relative ranking of characteristics of information material between states (Supplementary Material S7).

## 4. Discussion

This study investigated the knowledge, attitudes and behaviors regarding TBE vaccination and prevention of TBE and other TBDs such as Lyme borreliosis and tularemia among primary care physicians in private practices of the German southern states Bavaria and Baden-Wuerttemberg. Among our sample of 2321 physicians, almost all reported having adequate knowledge of TBE, TBE vaccination and other TBDs and offering TBE vaccinations.

In order for physicians to provide appropriate counselling for TBD prevention, including TBE vaccination, their own knowledge needs to be adequate. The role of practice staff should also not be underestimated with respect to the patients’ immunization counseling and decision making. The results among our sample indicate that almost all physicians perceived themselves as being adequately informed and aware of existing guidelines and recommendations. Nevertheless, awareness of the STIKO@RKI vaccination app, which offers up-to-date and evidence-based information on vaccination to healthcare professionals that provide vaccinations, was relatively low at 34%. Although similar mobile applications are available across other countries [28,29,30], to the best of our knowledge there have not been evaluations of health care providers’ awareness of them, nor quantifications of their effect. In addition, we did not assess whether the physicians’ and also their staff’s knowledge were, in fact, adequate. Studies among other samples of physicians in Italy [31], Serbia [32] and France [33,34] which tested specific knowledge of TBDs and their prevention and management showed that actual knowledge was only adequate in a limited proportion, suggesting that physicians might overestimate it [34]. As such, continued medical education programs, guidelines and public health campaigns and promotion of the STIKO@RKI vaccination app could increase and maintain knowledge among physicians and staff to ensure appropriate practices are being carried out.

A majority of our sample already used informational materials on TBE vaccination and TBDs. Nevertheless, a quarter indicated a (further) need for such materials. Traditional, analogue media, primarily posters and information flyers, were most popular among those materials already used and still needed. Specifically, physicians in our sample needed materials that are up-to-date, of high quality, easy to understand and independent of the pharmaceutical industry. Based on this information, we are currently developing materials for distribution and use among physicians and their patients.

It is reassuring that the proportion of primary care physicians in our sample who offer TBE vaccination and TBD prevention counselling was high, as this indicates accessibility, particularly considering costs are—as a rule—covered by health insurances in Germany. However, despite accessibility, the uptake of TBE vaccination is very limited and a substantial number of TBD cases, including TBE cases, are reported in these states each year [13]. Since a high proportion of TBE cases were not sufficiently vaccinated against the disease, the majority of those cases could likely have been prevented by vaccination [21]. Similarly, a household survey conducted among a representative sample of the general population in 20 European countries in 2020 showed that uptake of TBE vaccination in TBE-endemic countries varied highly from 7% in Romania to 81% in Austria, but was overall low at 22% [35]. According to this survey, physician recommendations appeared to be the most effective driver of TBE vaccination across both endemic and non-endemic countries.

In our sample, we found that a relatively limited proportion of physicians actively offered vaccination or provided information materials, suggesting that uptake heavily relies on patient request. There might be several explanations for why the proportion of physicians who actively offer vaccination is limited. These would include general high work pressure, lack of time during consultations booked for other health issues, inability to claim vaccination consultations if no vaccination is administered, having too many topics to discuss and this not being a priority, or not having an easy system available to record vaccinations. With respect to the last point, just over a quarter of those regularly checking vaccination status had a reminder system integrated in their practice’s software. A vaccination management system and information system, such as ImpfDocNE [36], might increase the active reminding and offering of vaccination by primary care physicians and increase vaccination uptake among patients [37]. Based on the results of a dissertation by Schuler, influenza vaccination coverage among chronically ill patients aged 18 to 89 years was increased three-fold to 80% and coverage for pneumococcal vaccination doubled to 65% after 4 years of using ImpfDocNE [38]. The study included data from 109 participating medical practices with 619,798 anonymised vaccination records of 133,559 patients. Other potential strategies to increase uptake might be an information campaign for primary care physicians to improve active offering or to increase patient requests. The use of incentives such as free provision of an immunisation management programme, higher payment for vaccinations and paying vaccination consultations separately might also be viable ways to increase active recommendation behavior among primary care physicians. Several studies have shown that the use of a vaccination reminder or recall system is an appropriate means of increasing vaccination rates [39]. Thereby, centralized approaches, that is, approaches using a state immunization information system, have been shown to be more effective and cost-effective compared to a practice-based approach [39]. Vaccination reminders can be sent in the form of text messages, emails or postcards, whereby the former has been shown particularly effective [39].

This study investigated physician-related factors of the provision of TBE vaccination and TBD prevention counselling. Even if knowledge of TBDs including TBE and active vaccination and counselling provision improves among primary care physicians, uptake might still be limited due to patient-related factors. Patient-related factors identified by studies that could be further researched and addressed to improve patient request and uptake of TBE vaccination and TBD prevention counselling include TBE risk perception, knowledge of TBE and benefits and risks of vaccination, including misinformation, awareness of vaccines (or a lack thereof, especially if not attending primary care physicians frequently), awareness of living in a TBE risk area, attitudes towards vaccination in general, barriers to healthcare access and health care seeking including stigmatization and general health-seeking behavior [20,22,35,40,41,42,43]. In Austria, there has been an annual national TBE awareness and vaccination campaign since 1980 for the general population [43]. Austria has the highest TBE vaccination uptake in Europe, and awareness among the population is high [37]. As a result, TBE cases have declined substantially [44].

Tularemia is rarely considered by primary care physicians when patients present with lymphadenitis and fever or other (often non-specific) symptoms of tularemia. Therefore, it can be assumed that tularemia is significantly underdiagnosed and underreported in Germany [45]. This is also reflected in the findings of our study: only 2% of participating physicians ever treated a tularemia patient, which was slightly higher in more rural versus more urban areas. Especially in areas with a high incidence of tularemia, doctors should therefore be informed about this rare and increasingly tick-borne zoonosis.

The results of our study appeared similar for primary care physicians in the two states. This resemblance could be the result of similarities in terms of personal and practice characteristics; their proximity; the fact that most districts in both states are classified as TBE risk areas; the uniform provision of information on and management of TBDs by the National Public Health Institute; and collaboration between respective regional public health institutes in terms of TBDs.

To the best of our knowledge, this study is the first large study on physician-related factors with respect to the prevention of TBDs, including TBE vaccination. We will directly use the results to create information education materials in order to improve primary care physicians’ counselling on TBE vaccination and other TBD prevention measures. While our study gathered valuable data, it is not without limitations. First, the response rate was limited. Nevertheless, a comparable rate would be expected based on many other studies conducted among similar samples of physicians in Germany [46,47,48,49]. We tried to make the survey as attractive as possible by limiting its length, including multiple-choice options and offering several possibilities for completion: on paper (with free-of-charge postage) and through computer, tablet or mobile phone (via a link or QR code). Second, there might have been selection or participation bias. We could have overestimated vaccination provision and knowledge if physicians who do not offer vaccinations or counselling on vaccination or other TBD prevention measures or who have less knowledge and experience were less likely to participate. The study sample might therefore not be representative of all primary care physicians in private practices in Baden-Wuerttemberg and Bavaria. Third, we did not test whether knowledge of TBDs and TBE vaccination was adequate nor whether treatment was appropriately conducted. Fourth and last, all measures were self-reported. Self-reporting might induce information bias due to social desirability bias. We, however, consider this type of bias likely to be minimal since the survey was anonymous and independently completed without oversight.

## 5. Conclusions

Self-reported vaccination provision and knowledge of TBE vaccination and TBDs was high in a sample of primary care physicians working in private practices in two large states in Germany with the highest incidence of TBDs. To improve uptake of TBE vaccination and TBD prevention measures to prevent TBDs among residents of these states, active offering of vaccinations and education could be increased, and additional, low-threshold information materials could be developed. Based on these results, we are currently developing various materials on TBDs and the prevention of TBDs, including TBE vaccination, in particular flyers and posters; these will be soon available for use by physicians during consultations.

## Figures and Tables

**Table 1:** Characteristics of primary care physicians participating in the TBD-Prev survey, Baden-Wuerttemberg and Bavaria, Germany, 24 May–30 September 2022.

		State		
Characteristic	Overall N = 2321	Baden-Wuerttemberg n = 1222	Bavaria n = 1067	Not Coded n = 32
**Online or paper questionnaire**				
Online	797 (34%)	431 (35%)	359 (34%)	7 (22%)
Paper	1524 (66%)	791 (65%)	708 (66%)	25 (78%)
**Gender**				
Female	1009 (44%)	510 (42%)	489 (46%)	10 (50%)
Male	1278 (56%)	700 (58%)	568 (53%)	10 (50%)
Not specified	15 (0.7%)	7 (0.6%)	8 (0.8%)	0 (0%)
**Age in years**				
≤30	0 (0%)	0 (0%)	0 (0%)	0 (0%)
31–40	169 (7.4%)	81 (6.7%)	87 (8.2%)	1 (5.3%)
41–50	504 (22%)	260 (21%)	238 (22%)	6 (32%)
51–60	864 (38%)	477 (39%)	382 (36%)	5 (26%)
>60	753 (33%)	393 (32%)	353 (33%)	7 (37%)
**Type of practice**				
Individual practice	1165 (51%)	606 (50%)	548 (53%)	11 (58%)
Joint or group practice	1075 (47%)	587 (49%)	480 (46%)	8 (42%)
Medical care center	11 (0.5%)	5 (0.4%)	6 (0.6%)	0 (0%)
Other	15 (0.7%)	7 (0.6%)	8 (0.8%)	0 (0%)
**Urban–rural level of practice (NUTS level 3)**				
Predominantly rural	494 (22%)	127 (10%)	367 (34%)	Missing
Intermediate	1030 (45%)	588 (48%)	442 (41%)	Missing
Predominantly urban	760 (33%)	503 (41%)	257 (24%)	Missing
**Medical speciality**				
General medicine/practitioner	1533 (66%)	746 (61%)	774 (73%)	13 (65%)
Internal medicine (primary care, without further specialisation)	339 (15%)	297 (24%)	38 (3.6%)	4 (20%)
Pediatrics	334 (14%)	127 (10%)	206 (19%)	1 (5.0%)
Other	100 (4.3%)	49 (4.0%)	49 (4.6%)	2 (10%)
**Offers travel medicine advice**	1863 (81%)	963 (80%)	884 (83%)	16 (80%)
**Regularly gets vaccinated against influenza**	1949 (85%)	1035 (85%)	890 (84%)	24 (80%)
**Vaccinated against TBE**	1937 (84%)	1026 (85%)	888 (84%)	23 (77%)

Column percentages may not add up to 100% due to rounding. Abbreviations: NUTS, nomenclature of territorial units for statistics; TBE, tick-borne encephalitis. There were missing data for gender (n = 19), age (n = 31), type of practice (n = 55), urban–rural level (n = 37), medical speciality (n = 15), travel medicine advice (n = 28), influenza vaccination (n = 18) and TBE vaccination (n = 21).

**Table 2:** Awareness of TBE guidelines and experience with tick-borne diseases among primary care physicians participating in the TBD-Prev survey, Baden-Wuerttemberg and Bavaria, Germany, 24 May–30 September 2022.

		State		
Characteristic	Overall N = 2321	Baden-Wuerttemberg n = 1222	Bavaria n = 1067	Not Coded n = 32
**Is aware of:**				
Current STIKO recommendations	2088 (91%)	1114 (92%)	955 (90%)	19 (100%)
STIKO@RKI vaccination app	776 (34%)	432 (36%)	340 (32%)	4 (21%)
TBE risk areas	2065 (90%)	1105 (92%)	944 (89%)	16 (84%)
Practice located in a TBE risk area	2255 (99%)	1199 (99%)	1037 (98%)	19 (100%)
**Has experience with:**				
Treating TBE	1110 (48%)	612 (50%)	488 (46%)	10 (53%)
Treating Lyme borreliosis	2203 (96%)	1162 (96%)	1023 (97%)	18 (95%)
Treating tularemia	55 (2.4%)	27 (2.2%)	28 (2.6%)	0 (0%)
Treating other TBDs	197 (8.6%)	96 (7.9%)	100 (9.4%)	1 (5.3%)
No experience with treating TBDs	60 (2.6%)	34 (2.8%)	26 (2.5%)	0 (0%)
**Perceives knowledge about risks and benefits of vaccination in general as adequate**	2260 (98%)	1198 (99%)	1033 (97%)	29 (94%)

Column percentages may not add up to 100% due to rounding. Abbreviations: STIKO, German Standing Committee on Vaccination; TBD, tick-borne diseases; TBE, tick-borne encephalitis. There were missing data for awareness of STIKO recommendations (n = 29), STIKO@RKI app (n = 38), TBE risk areas (n = 34) and practice located in TBE risk area (n = 34); experience with treating TBE (n = 30), Lyme borreliosis (n = 30) and tularemia (n = 30) or no experience (n = 30); and perceived knowledge or risks and benefits of vaccination (n = 12).

**Table 3:** Vaccination practices of primary care physicians participating in the TBD-Prev survey, Baden-Wuerttemberg and Bavaria, Germany, 24 May–30 September 2022.

		State		
Characteristic	Overall N = 2321	Baden-Wuerttemberg n = 1222	Bavaria n = 1067	Not Coded n = 32
**Provides vaccinations**	2226 (97%)	1189 (98%)	1019 (96%)	18 (95%)
**Provides TBE vaccinations**	2230 (97%)	1184 (97%)	1018 (96%)	28 (93%)
**Method(s) of offering vaccination consultations:**				
During initial consultation for new patients	1541 (67%)	829 (68%)	700 (66%)	12 (40%)
Regularly for all patients by actively approaching them	1469 (64%)	766 (63%)	683 (64%)	20 (67%)
At the active request of patients	1846 (80%)	964 (79%)	861 (81%)	21 (70%)
In the context of preventive medical check-ups	2049 (89%)	1082 (89%)	947 (89%)	20 (67%)
Through providing information materials	1351 (59%)	704 (58%)	633 (60%)	14 (47%)
Does not offer vaccination consultations	34 (1.5%)	14 (1.2%)	20 (1.9%)	0 (0%)
**Regularly checks vaccination status of patients**	2030 (89%)	1075 (89%)	930 (88%)	25 (81%)
**Reminds patients of due vaccinations**	1934 (84%)	1022 (84%)	888 (84%)	24 (80%)
**Among those who remind patients (N = 1934), uses reminder system**	538 (28%)	278 (28%)	254 (29%)	6 (26%)
**Reminds patients of due TBE vaccinations**	1817 (79%)	958 (79%)	837 (79%)	22 (73%)
**Among those who remind patients (N = 1817), method of reminding patients:**				
Card/sheet of paper with next vaccination	1240 (69%)	642 (68%)	585 (70%)	13 (59%)
Letter or postcard	77 (4.3%)	39 (4.1%)	36 (4.3%)	2 (9.1%)
Email	88 (4.9%)	34 (3.6%)	53 (6.4%)	1 (4.5%)
Telephone call	264 (15%)	117 (12%)	145 (17%)	2 (9.1%)
SMS (or WhatsApp, etc.)	36 (2.0%)	12 (1.3%)	24 (2.9%)	0 (0%)
Other methods	738 (41%)	384 (41%)	344 (41%)	10 (45%)
**Advises against TBE vaccination for:**				
Children	46 (2.2%)	23 (2.1%)	22 (2.3%)	1 (3.7%)
Persons with a medical contraindication	1841 (88%)	965 (88%)	855 (88%)	21 (78%)
Persons who are not exposed to ticks	453 (22%)	244 (22%)	201 (21%)	8 (30%)
Persons who do not live in a TBE risk area	371 (18%)	208 (19%)	156 (16%)	7 (26%)
Other persons	98 (4.7%)	49 (4.5%)	48 (5.0%)	1 (3.7%)

Column percentages may not add up to 100% due to rounding. Abbreviations: TBE, tick-borne encephalitis. There were missing data for provides vaccinations (n = 35), provides TBE vaccinations (n = 12), method of offering vaccination consultations (n = 17), checks vaccination status (n = 28), reminds patients of due vaccinations (n = 22), uses reminder system (n = 25), reminds patients of due TBE vaccinations (n = 20), method of reminding patients for TBE vaccination (n = 20), advises against vaccination for groups (n = 231).

## Data Availability

Data are not publicly available, but can be obtained from the corresponding authors upon reasonable request.

## Supplementary Materials

The following supporting information can be downloaded at: https://www.mdpi.com/article/10.3390/microorganisms11040961/s1, S1: TBD-Prev study questionnaire (original in German); S2: TBD-Prev study questionnaire (translated from German); S3: R Packages used for data cleaning, management, analysis and visualization; S4: Table on awareness of TBE guidelines and experience with tick-borne diseases stratified by urban-rural level of practice; S5: Figure on information needs among physicians who report not being adequately informed regarding the benefits and risk profile of vaccination; S6: Reasons for not offering TBE vaccination; S7: Characteristics of information materials about TBE vaccination and tick-borne diseases that are most important to physicians, stratified by need.
