# Supplementary material for: Knowledge, Attitudes and Behaviors regarding Tick-Borne Encephalitis Vaccination and Prevention of Tick-Borne Diseases among Primary Care Physicians in Bavaria and Baden-Wuerttemberg, Germany, May–September 2022
Source: Microorganisms. 2023 Apr 7;11(4):961. doi: 10.3390/microorganisms11040961 (PMC10146000; doi:10.3390/microorganisms11040961)
Supplement: Supplementary file 1 [file microorganisms-11-00961-s001.zip › microorganisms-2259057-supplementary.pdf]

Baden-Württemberg
MINISTERIUM FÜR SOZIALES, GESUNDHEIT UND INTEGRATION
LANDESGESUNDHEITSAMT

Bayerisches Landesamt für
Gesundheit und Lebensmittelsicherheit

SUPPLEMENT

“Knowledge, attitudes and behaviors regarding tick-borne encephalitis vaccination and prevention of tick-borne diseases among physicians in private practices in Bavaria and Baden-Wuerttemberg, Germany, May-September 2022”

Liza Coyer*†, Aylin Sogan-Ekinci*, Benedikt Greutélaers, Julia Kuhn, Franziska S. Saller, Jana Hailer, Stefanie Böhm, Rainer Brosch, Christiane Wagner-Wiening** and Merle M. Böhmer 1,6**†

* Shared first authorship
** Shared last authorship
† Corresponding authors, Dr. Liza Coyer, MSc PhD (liza.coyer@lgl.bayern.de), Dr. Merle Böhmer, MSc PhD (merle.boehmer@lgl.bayern.de)

S1. TBD-Prev study questionnaire (original in German)

TBD-Prev Studie

Zeckenübertragene Krankheiten, Zeckenprävention, FSME-Impfung

Impfakzeptanz und Management der Impfberatung zur FSME bei niedergelassenen Ärztinnen und Ärzten in der hausärztlichen Versorgung in Baden-Württemberg und Bayern

Sehr geehrte Kolleginnen und Kollegen,

im Jahr 2020 wurden in Baden-Württemberg und Bayern so viele Fälle von Frühsommer-Meningoenzephalitis (FSME) wie noch nie an die Gesundheitsämter gemeldet. Auch für die Lyme-Borreliose wurde ein Rekord an Erkrankungsfällen verzeichnet.

Generell steigen seit mehreren Jahren die Fallzahlen zeckenübertragener Krankheiten – insbesondere in Süddeutschland – an. Gesetztes Ziel in diesen besonders betroffenen Regionen ist es daher, die Bevölkerung durch die Vermittlung von Kenntnissen zu Zecken, zeckenübertragenen Krankheiten und der Aufklärung zu Möglichkeiten der Prävention zu sensibilisieren und das Risiko des Erwerbs dieser Krankheiten langfristig zu reduzieren. Im Rahmen der hausärztlichen Versorgung spielen Sie als niedergelassene Ärztin bzw. Arzt eine zentrale Rolle bei der Aufklärung und Beratung Ihrer Patienten(-innen) zu FSME und weiteren zeckenübertragenen Infektionen.

Mit dem Ziel, Strategien zur Erhöhung der FSME-Impfquoten abzuleiten sowie den Wissensstand zu FSME und weiteren zeckenübertragenen Krankheiten zu verbessern, haben das Landesgesundheitsamt Baden-Württemberg und das Bayerische Landesamt für Gesundheit und Lebensmittelsicherheit gemeinsam die vom Bundesministerium für Bildung und Forschung geförderte **TBD-Prev (Tick-Borne Disease Prevention) Studie** initiiert.

Mit beiliegendem Fragebogen möchten wir Sie im Rahmen dieser Studie zu FSME und anderen zeckenübertragenen Infektionen befragen. Mit Ihren Antworten können wir zum einen bestehende Wissenslücken zum Thema FSME sowie Impfhindernisse aus Ihrer, der ärztlichen Sicht identifizieren und zum anderen Ihren Bedarf an Informationsmaterialien zum Thema zeckenübertragene Erkrankungen und FSME-Impfung ermitteln.

Durch Ihre Teilnahme an der anonymen Befragung leisten Sie einen wichtigen Beitrag zur Prävention zeckenübertragener Krankheiten. Bitte unterstützen Sie uns, indem Sie den Fragebogen vorzugsweise digital ausfüllen. Den entsprechenden Link bzw. QR-Code sowie wichtige Hinweise zum Ausfüllen des Fragebogens finden Sie auf der umliegenden Seite. Alternativ kann der Fragebogen auch analog ausgefüllt und per beiliegendem, vorfrankiertem Rückumschlag an uns zurücksendet werden.

Herzlichen Dank für Ihre Unterstützung!

Ihr TBD-Prev Studienteam

Dr. Christiane Wagner-Wiening
Landesgesundheitsamt Baden-Württemberg
Tel.: 0711 904-39504
E-Mail: christiane.wagner-wiening@sm.bwl.de

Dr. Merle Böhmer
Bayerisches Landesamt für Gesundheit
und Lebensmittelsicherheit
Tel.: 09131-6808-5634
E-Mail: Merle.Boehmer@lgl.bayern.de

TBD-Prev Studie

Zeckenübertragene Krankheiten, Zeckenprävention, FSME-Impfung

Hinweise zum Ausfüllen des Fragebogens

Auf den folgenden 6 Seiten (beidseits bedruckt) finden Sie insgesamt 25 Fragen zu den folgenden Themenbereichen:

In **Block A** erfragen wir allgemeine Informationen zu Ihrer Praxis und Ihrer ärztlichen Tätigkeit, in **Block B** werden Fragen zu Ihrer Einstellung bzw. Ihrem Wissen zu zeckenübertragenen Erkrankungen, Impfen im Allgemeinen und im Speziellen zur FSME-Impfung gestellt.

Darüber hinaus interessiert uns Ihr Beratungsverhalten hinsichtlich zeckenübertragener Erkrankungen, Impfen und FSME-Impfung (**Block C**), sowie Ihr Bedarf an Informationsmaterialien zu dieser Thematik (**Block D**).

Bitte füllen Sie den Fragebogen vorzugsweise digital aus.

Über diesen Link
https://survey.lamapoll.de/TBE-Prev-Aerztebefragung

oder den nebenstehenden QR-Code
können Sie den Fragebogen digital ausfüllen.

+

Alternative: analog

Bitte füllen Sie den Fragebogen aus und senden Sie die drei ausgefüllten Blätter per beiliegendem, vorfrankiertem Rückumschlag an uns zurück.

Ausfüllhinweise:

Da die ausgefüllten Fragebögen von uns maschinell erfasst werden, möchten wir Sie bitten, die folgenden Hinweise zu beachten:

- ***Bitte markieren Sie so:*** ☒

  Nicht so:
- *Bitte verwenden Sie nach Möglichkeit einen **schwarzen Stift**. Vielen Dank!*
- *Auf allen Seiten des Fragebogens finden sich fünf sogenannte Justierpunkte (+), die für das maschinelle Einlesen notwendig sind.*

A. Allgemeine Angaben

1. Sind Sie als Ärztin/Arzt in einer niedergelassenen Praxis tätig?

☐ Ja ☐ Nein

1.1. In welcher Praxisform sind Sie tätig? (Einfachauswahl)

☐ Einzelpraxis
☐ Gemeinschaftspraxis bzw. Praxisgemeinschaft
☐ MVZ
☐ Andere

2. In welchem Land- bzw. Stadtkreis sind Sie tätig?
(Bitte das KFZ-Kennzeichen und Zuordnung Land- bzw. Stadtkreis angeben)

Kfz-Kennzeichen: | | | |

☐ Landkreis
☐ Stadtkreis

3. Bieten Sie reisemedizinische Beratung an?

☐ Ja ☐ Nein

4. In welcher Fachrichtung sind Sie tätig? (Einfachauswahl)

☐ Allgemeinmedizin
☐ Praktischer Arzt/praktische Ärztin
☐ Innere Medizin (hausärztliche Versorgung, ohne Schwerpunktbezeichnung)
☐ Kinder- und Jugendmedizin
☐ Andere

5. Geschlecht:

☐ Weiblich ☐ Männlich ☐ Divers ☐ keine Angabe

6. Zugehörigkeit zu Altersgruppe:

☐ ≤ 30 Jahre ☐ 31-40 Jahre ☐ 41-50 Jahre
☐ 51-60 Jahre ☐ > 60 Jahre ☐ keine Angabe

B. Einstellung / Wissen zu zeckenübertragenen Erkrankungen, Impfen und FSME-Impfung

7. Waren Ihnen <u>vor</u> Ausfüllen dieses Fragebogens die aktuellen Empfehlungen der Ständigen Impfkommission beim Robert Koch-Institut (STIKO) 2022 (veröffentlicht im Epidemiologischen Bulletin 4/2022) bekannt?

☐ Ja ☐ Nein

8. **War Ihnen <u>vor</u> Ausfüllen dieses Fragebogens *STIKO@rki* – die neu gestaltete Impf-App für Ärztinnen/Ärzte und Gesundheitsfachpersonal (verfügbar als Web-Version sowie als App für Android, iOS und Windows 10) – bekannt?**

☐ Ja ☐ Nein

9. **Waren Ihnen <u>vor</u> Ausfüllen dieses Fragebogens die aktuellen FSME-Risikogebiete in Deutschland (veröffentlicht im Epidemiologischen Bulletin 9/2022 sowie verfügbar auf der Internetseite des RKI) bekannt?**

☐ Ja ☐ Nein

10. **War Ihnen <u>vor</u> Ausfüllen dieses Fragebogens bekannt, ob der Land- bzw. Stadtkreis in dem Sie praktizieren, ein FSME-Risikogebiet ist?**

☐ Ja ☐ Nein

11. **Haben Sie in Ihrer ärztlichen Tätigkeit bereits die folgenden zeckenübertragenen Erkrankungen behandelt?** (Mehrfachauswahl möglich)

☐ FSME
☐ Lyme-Borreliose
☐ Tularämie
☐ Andere zeckenübertragene Krankheit
☐ Nein, ich habe keine zeckenübertragene Krankheit behandelt

12. **Führen Sie in Ihrer Praxis Schutzimpfungen (allgemein) durch?**

☐ Ja ☐ Nein

12.1. Wenn nein, warum nicht? (Mehrfachauswahl möglich)

☐ Logistische Schwierigkeiten
☐ Hoher Aufklärungsaufwand
☐ Non-Compliance seitens der Patienten
☐ Bedenken bezüglich der Sicherheit von Impfungen
☐ Geringes Risiko beim Durchlaufen impfpräventabler Erkrankungen
☐ Zweifel an der Wirksamkeit von Impfungen
☐ Andere Gründe

13. Führen Sie in Ihrer Praxis Schutzimpfungen gegen FSME durch?

☐ Ja ☐ Nein

13.1. Wenn nein, warum nicht? (Mehrfachauswahl möglich)

- ☐ Logistische Schwierigkeiten
- ☐ Hoher Aufklärungsaufwand
- ☐ Non-Compliance seitens der Patienten
- ☐ Bedenken bezüglich der Sicherheit der FSME-Impfung
- ☐ Geringes gesundheitliches Risiko durch eine FSME-Infektion
- ☐ Zweifel an der Wirksamkeit der FSME-Impfung
- ☐ Andere Gründe

14. Fühlen Sie sich selbst als Ärztin/Arzt hinsichtlich des Nutzens und Risikoprofils von Schutzimpfungen (allgemein) ausreichend informiert?

☐ Ja ☐ Nein

14.1. Wenn nein, welchen Informationsbedarf haben Sie?
(Mehrfachauswahl möglich)

- ☐ Fortbildungen zum Thema Schutzimpfungen
- ☐ Fortbildungen zum Thema impfpräventable Erkrankungen
- ☐ Informationen zur aktuellen Situation impfpäventabler Erkrankungen in Ihrem Stadt-/Landkreis
- ☐ Informationen zur aktuellen Verfügbarkeit von Impfstoffen
- ☐ Andere

15. Würden Sie von sich selbst sagen, dass Sie gegenüber Zecken exponiert sind?

☐ Ja ☐ Nein

16. Sind Sie selbst gegen FSME geimpft?

☐ Ja ☐ Nein

17. Lassen Sie sich regelmäßig gegen Influenza impfen?

☐ Ja ☐ Nein

C. Beratungsverhalten hinsichtlich zeckenübertragener Erkrankungen, Impfen und FSME-Impfung

18. Bei welchem Anlass führen Sie in Ihrer Praxis Impfberatungen durch?
(Mehrfachauswahl möglich)

- ☐ Bei neuen Patientinnen/Patienten im Erstgespräch
- ☐ Regelmäßig für alle Patientinnen/Patienten durch aktive Ansprache
- ☐ Auf aktive Nachfrage der Patientinnen/Patienten
- ☐ Im Rahmen von Vorsorgeuntersuchungen
- ☐ Wir informieren über Hinweise wie Flyer, Plakate, o.ä.
- ☐ Gar nicht

19. Überprüfen Sie den Impfstatus Ihrer Patientinnen/Patienten regelmäßig?

☐ Ja ☐ Nein

20. Erinnern Sie Ihre Patientinnen/Patienten <u>allgemein</u> an fällige Impfungen?

☐ Ja ☐ Nein

20.1. Wenn ja, nutzen Sie ein in Ihre Praxissoftware integriertes Erinnerungssystem?

☐ Ja ☐ Nein

21. Erinnern Sie Ihre Patientinnen/Patienten an fällige <u>FSME</u>-Impfungen?

☐ Ja ☐ Nein

21.1. Wenn ja, in welcher Form erinnern Sie Ihre Patientinnen/Patienten an fällige FSME-Impfungen? (Mehrfachauswahl möglich)

- ☐ Die Patientinnen/Patienten bekommen ein Kärtchen/Zettel mit dem nächsten Impftermin
- ☐ per Brief oder Postkarte
- ☐ per E-Mail
- ☐ per Telefonanruf
- ☐ per SMS (WhatsApp o.ä.)
- ☐ Andere

22. Welchen Personengruppen würden Sie von einer FSME-Impfung abraten? (Mehrfachauswahl möglich)

☐ Kindern, generell
☐ Personen, bei denen medizinische Gründe gegen die Impfung sprechen
☐ Personen, die nicht zeckenexponiert sind
☐ Personen, die nicht in einem FSME-Risikogebiet wohnen
☐ Andere

22.1. Wenn Sie Kindern generell von einer FSME-Impfung abraten, aus welchen Gründen? (Mehrfachauswahl möglich)

☐ Kinder erkranken meist nicht schwer an FSME
☐ Kinder erkranken meist nur asymptomatisch
☐ Kinder sind generell weniger gegenüber Zecken exponiert
☐ Bei Kindern überwiegt das Risiko der Impfung den Nutzen
☐ Andere

23. Nutzen Sie Informationsmaterialien zur FSME-Impfung und zeckenübertragenen Erkrankungen in Ihrer Praxis?

☐ Ja ☐ Nein

23.1. Welches Informationsmaterial bzw. welche Aufklärungsmedien nutzen Sie? (nur, wenn 23. „Ja“) (Mehrfachauswahl möglich)

☐ Poster
☐ Informationsflyer
☐ Aufkleber
☐ Aufsteller
☐ Videoclips
☐ Posts auf Sozialen Medien
☐ Die eigene Homepage
☐ Andere

D. Bedarf an Informationsmaterialien

24. Haben Sie (weiteren) Bedarf an Informationsmaterialien und Aufklärungsmedien zur FSME-Impfung und zeckenübertragenen Erkrankungen?

☐ Ja

☐ Nein

+ +

24.1 Für welche Art von Informationsmaterialien und Aufklärungsmedien besteht Bedarf?
(nur, wenn 24. „Ja") (Mehrfachauswahl möglich)

☐ Poster
☐ Informationsflyer
☐ Aufkleber
☐ Aufsteller für Ihre Praxis
☐ Videoclips
☐ Foliensätze (z.B. PowerPoint) als Mustervorträge
☐ Infographiken (z.B. für Social Media Auftritt)
☐ Andere

***25.* Welche Eigenschaften sind Ihnen bei Informationsmaterialien und Aufklärungsmedien zur FSME-Impfung und zeckenübertragenen Erkrankungen am wichtigsten?**
Bitte geben Sie ein Ranking ab. *Platzierung (A=am wichtigsten ➡ I=am unwichtigsten)*
HINWEIS: ***Jeder Buchstabe darf nur einmal angekreuzt werden***

Mir ist am wichtigsten, dass Informationsmaterialen und Aufklärungsmedien …

+

Rang:	A	B	C	D	E	F	G	H	I
… pharmaunabhängig sind.	☐	☐	☐	☐	☐	☐	☐	☐	☐
… qualitätsgesichert sind.	☐	☐	☐	☐	☐	☐	☐	☐	☐
… kostenlos sind.	☐	☐	☐	☐	☐	☐	☐	☐	☐
... aktuell, also auf dem aktuellen Wissensstand sind.	☐	☐	☐	☐	☐	☐	☐	☐	☐
… leicht verständlich sind.	☐	☐	☐	☐	☐	☐	☐	☐	☐
… zielgruppenspezifisch sind (z.B. angepasst an die Altersgruppe).	☐	☐	☐	☐	☐	☐	☐	☐	☐
… leicht zugänglich sind.	☐	☐	☐	☐	☐	☐	☐	☐	☐
… in verschiedenen Sprachen verfügbar sind.	☐	☐	☐	☐	☐	☐	☐	☐	☐
… andere	☐	☐	☐	☐	☐	☐	☐	☐	☐

Herzlichen Dank für Ihre Teilnahme!

+ +

S2. TBD-Prev study questionnaire (translated from German)

A. General information

1. Are you a physician in a private practice (i.e. general practioner)?

☐ Yes ☐ No

1.1. In which type of practice do you work (Single choice)

☐ Individual practice
☐ Joint practice or group practice
☐ Medical care centre („MVZ“)
☐ Other

2. In which county or city do you work?
(Please indicate the license number and county or city)

Kfz-Kennzeichen: ☐☐☐ ☐ Landkreis ☐ Stadtkreis

3. Do you offer travel medicine consulations?

☐ Yes ☐ No

4. In which specialization do you work? (Single choice)

☐ General medicine
☐ General practioner/physician
☐ Internal Medicine (primary care, without specialty designation)
☐ Pediatric and/or adolescent medicine

5. Gender:

☐ Female ☐ Male ☐ Non-binary ☐ Not specified

6. Age group:

☐ ≤ 30 years ☐ 31-40 years ☐ 41-50 years
☐ 51-60 years ☐ > 60 years ☐ Not specified

B. Attitude and knowledge about tick-borne diseases, vaccination, and TBE vaccination

7. Were you aware of the current recommendations of the Standing Committee on Vaccination at the Robert Koch Institute (STIKO) 2020/2021 (published in Epidemiological Bulletin 34/2020) before completing this questionnaire?

☐ Yes ☐ No

+ +

8. **Were you aware of the STIKO@rki (the newly designed vaccination app for physicians and health professionals, which is available as a web version and app for Android, iOS and Windows 10) before completing this questionnaire?**

☐ Yes ☐ No

9. **Were you aware of the current TBE risk areas in Germany (published in Epidemiological Bulletin 9/2021 and available on the RKI website) before completing this questionnaire?**

☐ Yes ☐ No

10. **Were you aware whether the rural or urban district in which you practice is a TBE risk area before completing this questionnaire?**

☐ Yes ☐ No

11. **Have you already treated tick-borne diseases in your medical practice?** (Multiple selections possible)

☐ No

☐ TBE

☐ Lyme borreliosis +

☐ Tularemia

☐ Other

12. **Do you provide vaccinations in your practice?**

☐ Yes ☐ No

12.1. If not, why not? (Multiple selections possible)

☐ Logistical difficulties

☐ High effort for clarification

☐ Non-compliance of the patients

☐ Concerns about the safety of the vaccinations

☐ Low health risk of vaccine-preventable diseases

☐ Doubts about the effectiveness of vaccinations

☐ Other reasons

13. Do you provide protective vaccinations against TBE in your practice?

☐ Yes ☐ No

13.1. If not, why not? (Multiple selections possible)

☐ Logistical difficulties
☐ High effort for clarification
☐ Non-compliance on the part of patients
☐ Concerns about the safety of TBE vaccination
☐ Low health risk from TBE infection
☐ Doubts about the effectiveness of TBE vaccination
☐ Other reasons

14. As a physician, do you feel that you yourself are adequately informed regarding the benefits and risk profile of vaccination?

☐ Yes ☐ No

14.1. If not, what information needs do you have?
(multiple selections possible)

☐ Continuing education on the topic of protective vaccinations
☐ Further training on the topic of vaccine-preventable diseases
☐ Information on the current situation of vaccine-preventable diseases in your county/city
☐ Information on the current availability of vaccines
☐ Other

15. Would you say of yourself that you are exposed to ticks?

☐ Yes ☐ No

16. Have you been vaccinated against TBE?

☐ Yes ☐ No

17. Do you get vaccinated against influenza regularly?

☐ Yes ☐ No

C. Counselling behavior regarding tick-borne diseases, vaccination and TBE vaccination

18. How do you conduct vaccination consultations in your practice? (Multiple selections possible)

- ☐ For new patients during the initial consultation
- ☐ Regularly for all patients by actively approaching them
- ☐ At the active request of patients
- ☐ In the context of preventive medical check-ups
- ☐ We provide information via notices such as flyers, posters, etc.
- ☐ Not at all

19. Do you regularly check the vaccination status of your patients?

☐ Yes ☐ No

20. Do you generally remind your patients of vaccinations that are due?

☐ Yes ☐ No

20.1. If yes, do you use a reminder system integrated in your practice software?

☐ Yes ☐ No

21. Do you remind your patients when TBE vaccinations are due?

☐ Yes ☐ No

21.1. If yes, in what way do you remind your patients when TBE vaccinations are due? (Multiple selections possible)

- ☐ Patients are given a card/sheet of paper with the next vaccination
- ☐ By letter or postcard
- ☐ By E-Mail
- ☐ By telephone call
- ☐ By SMS (WhatsApp etc.)
- ☐ Other

22. Which groups of people would you advice against getting a TBE vaccination? (Multiple selections possible)

- ☐ Children, in general
- ☐ Persons with a medical contradiction
- ☐ Persons who are not exposed to ticks
- ☐ Persons who do not live in a TBE risk area
- ☐ Other

22.1. If you generally advise against TBE vaccination for children, for what reasons? (Multiple selections possible)

- ☐ Children usually do not get severly ill with TBE
- ☐ Children usually fall ill only asymptomatically
- ☐ Children are generally less exposed to ticks
- ☐ The risk of vaccination outweighs the benefit for children
- ☐ Other

23. Do you use information materials about TBE vaccination and tick-borne diseases in your practice?

☐ Yes ☐ No

23.1. If yes, which information materials or educational media do you use? (Only if 23 „Ja“) (Multiple selections possible)

- ☐ Poster
- ☐ Information flyer
- ☐ Sticker
- ☐ Posts on social media
- ☐ Video clips
- ☐ Own homepage
- ☐ Other

D. Need for information materials

24. Do you have (further) need for information materials and educational media on TBE vaccination and tick-borne diseases?

- ☐ Yes
- ☐ No

24.1. For what kind of information materials and educational media is there a need? (Only when 24. „Ja“) (Multiple selections possible)

☐ Poster

☐ Information flyer

☐ Sticker

☐ Display stand for your practice

☐ Videoclips

☐ Slide sets (PowerPoint) as sample presentations

☐ Infographics (e.g. for social media practice)

☐ Other

25. **What characteristics are most important to you in information materials and educational media about TBE vaccination and tick-borne diseases?**
Please provide a ranking *(A=most important ➧ I=least important)*
HINWEIS: ***Each letter may only be selected once***

It is most important to me that information materials and educational media …

Rang:	A	B	C	D	E	F	G	H	I
… are independent of pharma.	☐	☐	☐	☐	☐	☐	☐	☐	☐
… are quality-assured.	☐	☐	☐	☐	☐	☐	☐	☐	☐
… are free of charge.	☐	☐	☐	☐	☐	☐	☐	☐	☐
... are up-to-date, i.e. at the current state of knowledge.	☐	☐	☐	☐	☐	☐	☐	☐	☐
… are easy to understand.	☐	☐	☐	☐	☐	☐	☐	☐	☐
… are specific to the target group (e.g. adapted to the age group).	☐	☐	☐	☐	☐	☐	☐	☐	☐
… are easily accessible.	☐	☐	☒	☐	☐	☐	☐	☐	☐
… are available in different languages.	☐	☐	☐	☐	☐	☐	☐	☐	☐
… other	☐	☐	☐	☐	☐	☐	☐	☐	☐

Thank you very much for your participation!

S3. R Packages used for data cleaning, management, analysis and visualization

tidyverse

lubridate

rio

readr

janitor

flextable

ggplot2

purr

writexl

gtsummary

expss

matrixStats

broom

labelled

S4. Table. Awareness of TBE guidelines and experience with tick-borne diseases among physicians participating in the TBD-Prev survey, stratified by urban-rural level of practice Baden-Wuerttemberg and Bavaria, Germany, 24 May 2022-30 September 2022.

		Urban-rural level of practice		
**Characteristic**	**Overall**<br>**N = 2,284***	**Predominantly rural**<br>**n = 494**	**Intermediate**<br>**n = 1,030**	**Predominantly urban**<br>**n = 760**
**Is aware of:**				
Current STIKO recommendations	2,064 (91%)	459 (94%)	923 (90%)	682 (90%)
STIKO@RKI vaccination app	769 (34%)	154 (31%)	355 (35%)	260 (35%)
TBE risk areas	2,044 (90%)	442 (90%)	929 (91%)	673 (90%)
Practice located in a TBE risk area	2,231 (99%)	482 (99%)	1,009 (99%)	740 (98%)
**Has experience with:**				
Treating TBE	1,100 (49%)	275 (56%)	541 (53%)	284 (38%)
Treating Lyme borreliosis	2,180 (96%)	479 (98%)	982 (96%)	719 (95%)
Treating tularemia	55 (2.4%)	17 (3.5%)	26 (2.5%)	12 (1.6%)
Treating other tick-borne diseases	196 (8.6%)	54 (11%)	84 (8.2%)	58 (7.7%)
No experience with treating tick-borne diseases	60 (2.6%)	8 (1.6%)	25 (2.4%)	27 (3.6%)
**Perceives knowledge about risks and benefits of vaccination in general as adequate**	60 (2.6%)	479 (97%)	1,006 (98%)	742 (98%)

Abbreviations: STIKO, German Standing Committee on Vaccination; TBE, tick-borne encephalitis. *There were missing data for urban-rural level of practice (n=37), awareness of STIKO recommendations (n=29), STIKO@RKI app (n=38), TBE risk areas (n=34), practice located in TBE risk area (n=34), experience with treating TBE (n=30), Lyme borreliosis (n=30), tularemia (n=30), no experience (n=30), perceived knowledge or risks and benefits of vaccination (n=12).

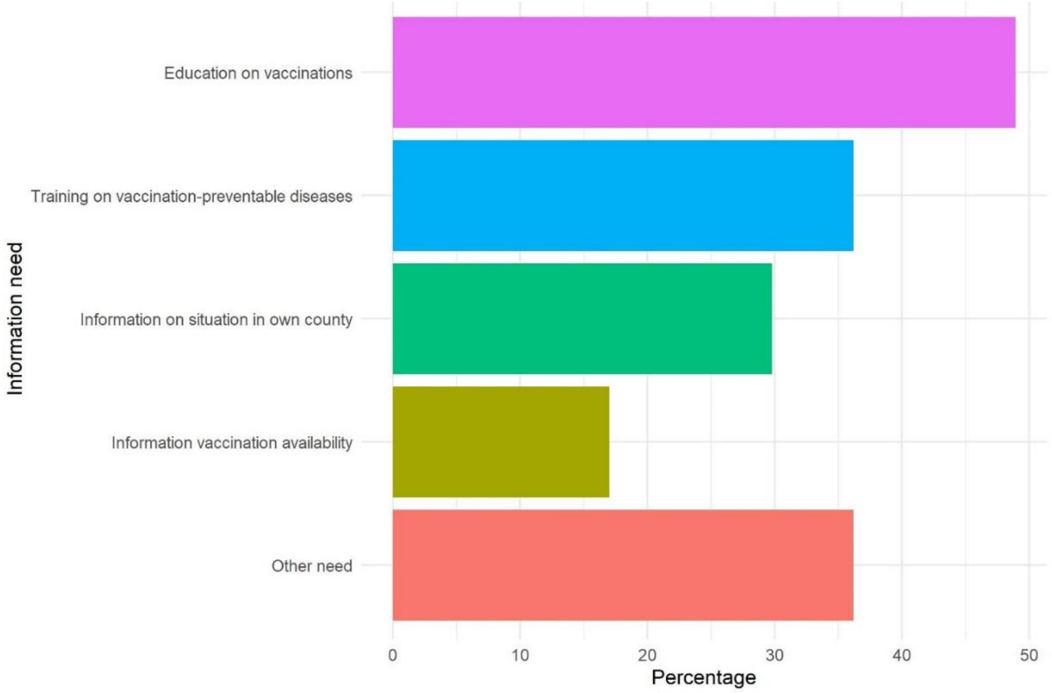

S5. Figure. Information needs among physicians who report not being adequately informed regarding the benefits and risk profile of vaccination (N=47), TBD-Prev survey, Germany, 24 May 2022-30 September 2022.
There were in total 49 physicians without adequate knowledge, but 2 did not complete the question on information need.

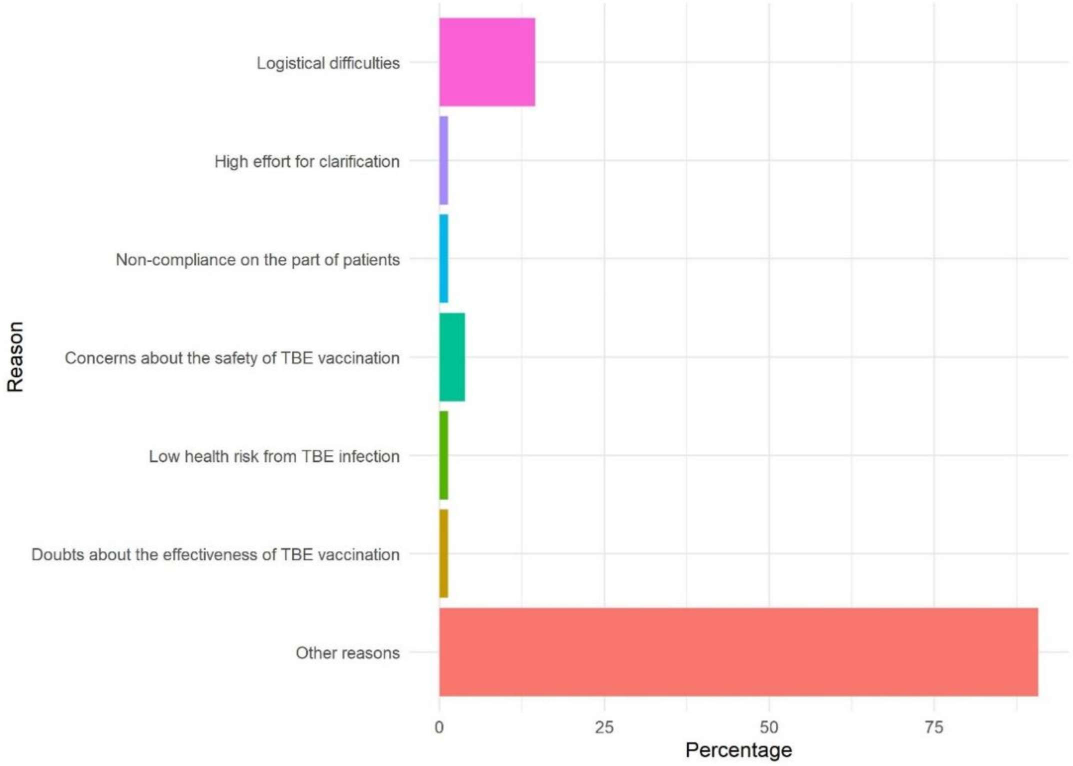

S6. Figure. Reasons for not offering TBE vaccination among 76 physicians participating in the TBD-Prev survey, Baden-Wuerttemberg and Bavaria, Germany, 24 May 2022-30 September 2022.
There were in total 79 physicians who did not offer TBE vaccinations, but 3 did not complete the question on reasons.

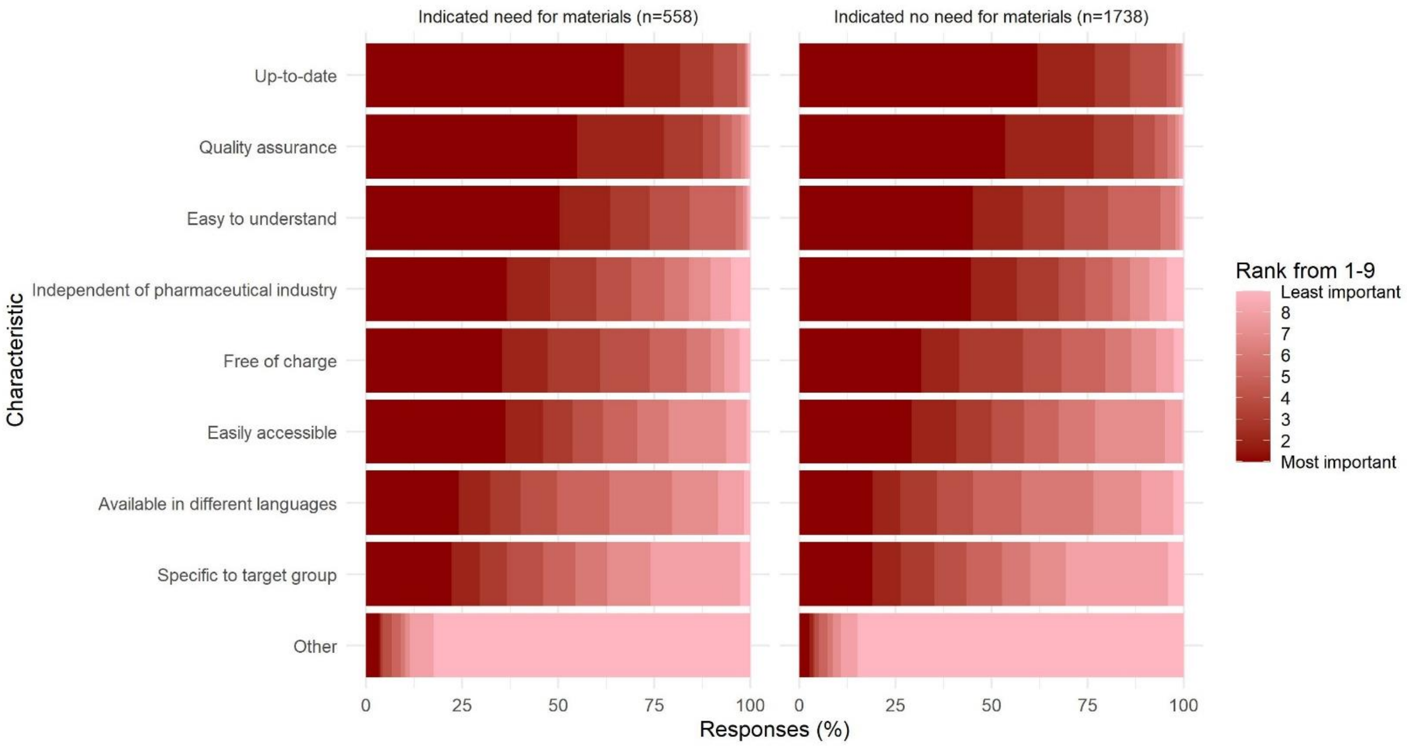

S7. Figure. Characteristics of information materials about TBE vaccination and tick-borne diseases that are most important to physicians (N=2,242), stratified by need, TBD-Prev survey, Germany, 24 May 2022-30 September 2022.

Physicians were asked to rank characteristics from 1 (most important) to 9 (least important). Of 2,321 participating physicians, 79 did not answer this question. 25 participants did not answer the question on need for materials and were also excluded from the graph.
